# Two-year follow-up of varus progression in young professional football players

**DOI:** 10.1186/s13102-026-02035-7

**Published:** 2026-09-11

**Authors:** Hannah Gablac, Lars Schwalm, Peter Behrendt, Michael Hoffmann, Matthias Krause, Karl-Heinz Frosch, Götz Welsch, Dimitris Dalos

**Affiliations:** 1 Muskuloskelettales Universitätszentrum München, Ludwig-Maximilians- Universität München, Munich, Germany; 2 UKE Athleticum - Center for Athletic Medicine, University Medical Center Hamburg-Eppendorf, Hamburg, Germany; 3 Institute of Interdisciplinary Exercise Science and Sports Medicine, MSH Medical School Hamburg, Hamburg, Germany; 4 Department of Trauma and Orthopaedic Surgery, University Medical Center Hamburg-Eppendorf, Hamburg, Germany; 5 Department of Trauma Surgery, Orthopaedics and Sports Traumatology, BG Hospital Hamburg, Hamburg, Germany; 6 Department of Trauma and Orthopaedic Surgery, University Medical Center Schleswig-Hohlstein, Kiel, Germany; 7 Sanatorium Dr. Schenk GmbH, Montafonerstraße 29, Schruns, 6780 Austria

**Keywords:** Leg axis, Football, Soccer, Genu varum, Osteoarthritis, Bow legs

## Abstract

**Purpose:**

Professional football players have an increased prevalence of genu varum, which is a risk factor for developing knee osteoarthritis. It remains unclear at what stage of adolescence varus progression is most pronounced and which factors are associated with this progression. Therefore, this study aimed to investigate the factors associated with varus progression over a two-year observation period in a selected cohort of adolescent male professional academy football players.

**Methods:**

A total of 31 male youth academy football players aged 12–17 years who demonstrated varus progression during the oberservation period were included. The static mean mechanical femorotibial axis angle (MAA) of both legs was measured using a two-dimensional and marker-based method (DIERS leg axis analysis system). Players’ age, height, BMI and dominant kicking leg were also assessed. Measurements were performed in 2021 and after a 24-month observation period. Changes and correlations over the observation period were statistically analyzed using SPSS.

**Results:**

Age-related differences were observed in the extent of MAA: the youngest athletes had the lowest MAA at baseline (*p* = 0.049), which increased more over the following two years than the older players. Greater increases in height during the observation period (*p* = 0.008), low initial height (*p* = 0.035), BMI (*p* = 0.043) and less varus deformity (*p* = 0.017) were found to be significant factors associated with an increase in MAA.

**Conclusion:**

Within this selected cohort, the increase in MAA over the two-year observation period was most pronounced in the younger athletes. Greater increases in height and associated weight gain were the variables most strongly associated with increasing varus alignment during the observation period. These findings may help to inform the timing of future preventive strategies, although their effectiveness for influencing leg-axis development remains to be established.

**Supplementary Information:**

The online version contains supplementary material available at https://doi.org/10.1186/s13102-026-02035-7.

## Introduction

A correlation between the practice of bone-strengthening sport during the growth phase and an associated progression of bowlegs (varus deformity) in adolescent athletes has been suggested [1–4]. The increase in varus deformity has been observed particularly in male football players who, at a young age, undergo increased training intensity and frequency over an extended period [1, 5–9] Numerous studies have demonstrated a correlation between varus deformity and the resulting cartilage degeneration, such as loss of tissue anisotropy, reduced cartilage water content and increased water mobility [10–12]. The changes seemed to be most pronounced in the medial tibiofemoral joint [13, 14]. Thus, varus deformity is thought to be a deciding risk factor for medial-sided osteoarthritis as it shifts the weight bearing line to the medial compartment [4, 15–18]. Cartilage defects coupled with sports-related knee injuries, can lead to osteoarthritis, which already has an above-average incidence in footballers and doubles in the presence of varus malalignmen [6, 19]. It remains unclear at what point in the athlete’s development the maximum varus deformity occurs and when the actual increase in MAA (mechanical femorotibial axis angle) itself is greatest. It has been suggested that the time around peak height velocity (PHV) may represent a vulnerable developmental period for leg-axis changes. However, PHV was not directly assessed in the present study and is therefore discussed only as a theoretical framework derived from previous literature. Furthermore, the influence of individual intrinsic risk factors such as age, height, weight and body mass index (BMI) is unclear. The gold standard for examining the leg axis is an X-ray of the entire leg [1]. As this cannot be carried out routinely without clinical indication due to the radiation exposure and the young age of the athlete, there are also radiation-free measurement methods for assessing the leg axis which show comparable results to the radiological gold standard [20].

The aim of the present study was to investigate factors associated with varus progression over a two-year observation period in a selected cohort of adolescent male academy football players. Furthermore, age-related differences in the progression of the mechanical femorotibial axis angle (MAA) were examined. It was hypothesized that greater increases in MAA would be associated with greater increases in height and would therefore tend to occur in younger athletes, whereas the greatest varus alignment would be observed in older athletes.

## Methods

The study was reviewed by the Ethics Committee of the local Medical Association (review number: PV5923) and conforms to the ethical standards of the 1964 Declaration of Helsinki.

### Study population

All participants were recruited from the Under-13 to Under-21 youth teams of a German professional football club academy. All participants had regularly participated in professionally supervised football-specific and athletic training as part of the youth academy for at least four years. The cohort therefore represents highly trained male academy players competing within the structured development programme of a professional football club.

Initial measurements were performed in 2021, and follow-up measurements were carried out in 2023 in 46 athletes. Exclusion criteria were acute knee injuries (*n* = 1), previous surgical correction of lower-limb malalignment (*n* = 1), and absence of varus progression during the observation period (*n* = 13). The latter criterion was applied because the objective of this study was to investigate factors associated with varus progression over time. This selection criterion should be considered when interpreting the findings, as it may limit their generalisability to the broader population of youth football players. A total of 31 athletes with a mean age of 14 ± 2 years were included in the study. The athletes were divided into three groups: *young adolescents*, *mid-adolescents*, and *older adolescents* according to age. Each age group comprised a two-year age range: 12–13 years (*n* = 11), 14–15 years (*n* = 11) and 16–17 years (*n* = 9) years at the first measurement in 2021. It should be noted that each group is two years older at the follow-up measurement in 2023, but retains its original group name in this paper for ease of understanding.

### Leg axis analysis

The leg axis analysis was performed as previously described by Ossendorff et al. and Dalos et al. [20, 21]. The validity of the DIERS Leg Axis System has been demonstrated in comparison with long-standing radiographs, which remain the gold standard for lower-extremity alignment assessment. Ossendorff et al. reported good agreement between both methods, supporting the use of the DIERS system as a reliable and radiation-free alternative for repeated measurements, particularly in young athletic populations, where minimizing radiation exposure is desirable [20]. Both legs of each athlete were measured and analyzed using the two-dimensional and marker-based DIERS Leg Axis System (DIERS international, Schlangenbad, Germany) [20]. All measurements were performed according to the manufacturer’s standardised protocol as closely as possible. Participants stood upright with both feet positioned in parallel and with symmetrical weight bearing. A single measurement per participant and time point was used for the analyses. Repeat measurements were only performed in the event of technical problems, such as displacement of marker reference points during data acquisition. For the primary analyses, the mean MAA of both legs was used to characterise overall lower-limb alignment and to minimise the influence of side-specific variability. Side-specific analyses and the potential influence of the dominant kicking leg were additionally explored.

**Fig. 1:**
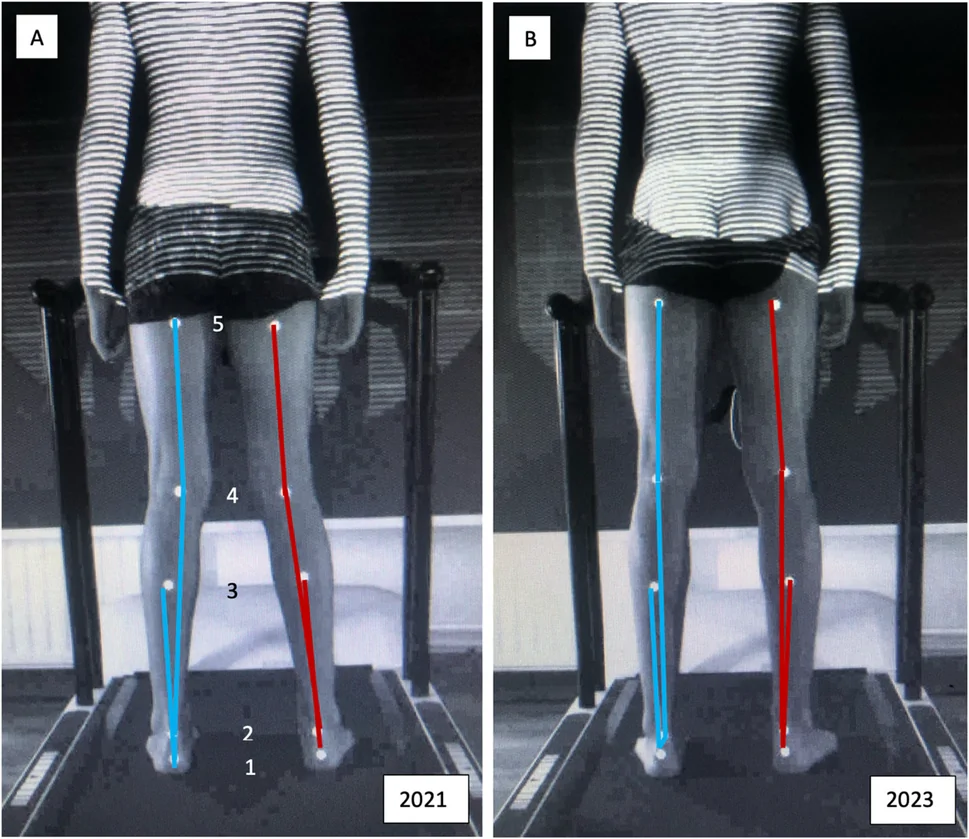
Representative case of an increased varusalignment measured by a camera-based analysis system in 2021 (**A**) and 2023 (**B**) showing the two-dimensional video documentation of the leg axis geometry (DIERS leg axis posterior showing the mechanical femorotibial axis angle (MAA) under static conditions. The MAA is formed between the mechanical axis of the femur and tibia as deviations in the mechanical leg axis. Defined landmarks include the lower (1) and upper (2) edges of the calcaneus, the intersection of the two gastrocnemius muscle bellies (3), the middle of the popliteal flexion crease (4) and centrally below the gluteal fold. [via: DIERS formetric 4D System, DIERS leg axis accessory of DIERS formetric 4D System, DIERS international, Schlangenbad, Germany]

The system is based on a two-dimensional video recording of the leg axis geometry in the frontal plane recorded by a posterior leg axis camera. For the former, the measurement was performed in an upright, neutral body position with the athletes fully loaded and wearing only underwear and no shoes. At the start of the study, five reflective tracking markers were placed on the skin of both legs at defined anatomical landmarks according to the manufacturer’s instructions: on the soft tissue just above and just below the calcaneus, on the tendinous insertion of the medial and lateral head of the gastrocnemius muscle, in the middle of the popliteal fossa and just below the gluteal half in the middle of the back of the thigh (Fig. 1). The correct position of the marked athletes on the treadmill was ensured by a line with both feet aligned in parallel. The recording unit of the DIERS Leg Axis System was placed centrally and orthogonally behind the treadmill and registered the reflection of the illuminated tracking markers on the participants’ skin in the frontal plane and derived the mechanical leg axis (MAA = mechanical femorotibial angle between the axis of the mechanical femur and the tibia) from the connecting lines (femur: 4 and 5, tibia: 2 and 4) between the individual skin markers. Negative values (MAA < 180°) were considered valgus and positive values (MAA > 180°) varus [20, 22] Data were recorded, processed and exported using the system’s own software.

### Data analysis

Means, standard deviations, and 95% confidence intervals for leg axis and demographic data in 2021 and 2023 were calculated for all 31 athletes, as well as changes in varus alignment and demographic variables over the observation period. To assess the influence of chronological age, the cohort was divided into three age groups spanning two years each: young (12–13 years, *n* = 11), mid-adolescent (14–15 years, *n* = 11), and older adolescent athletes (16–17 years, *n* = 9).

In accordance with the study hypothesis, chronological age at baseline and change in height over the 24-month observation period (ΔHeight) were defined a priori as the primary predictors of changes in MAA. Additional variables, including body weight, BMI, baseline MAA, and dominant kicking leg, were analysed as secondary exploratory factors.

Statistical analyses were performed using SPSS (IBM Corp., Armonk, NY, USA) with a significance level of *p* < 0.05. No a priori sample size calculation was performed, as this study was designed as an observational follow-up investigation including all eligible players from a single professional football academy. Consequently, the sample size was determined by the available cohort and should be considered when interpreting the statistical analyses, particularly the multivariable models.

Pearson correlation coefficients were calculated to examine associations between changes in MAA and individual demographic and anthropometric variables. Simple linear regression analyses were subsequently performed to investigate the relationship between individual predictors and changes in MAA. The primary multivariable model included age at baseline and ΔHeight. Additional exploratory multivariable analyses provided in the Supplementary Material.

## Results

Among the 31 included athletes, the mean MAA increased from 3.13 ± 3.40° at baseline (2021) to 6.11 ± 3.15° at follow-up (2023), corresponding to a mean increase of 2.89 ± 1.50° over the 24-month observation period.

**Fig. 2:**
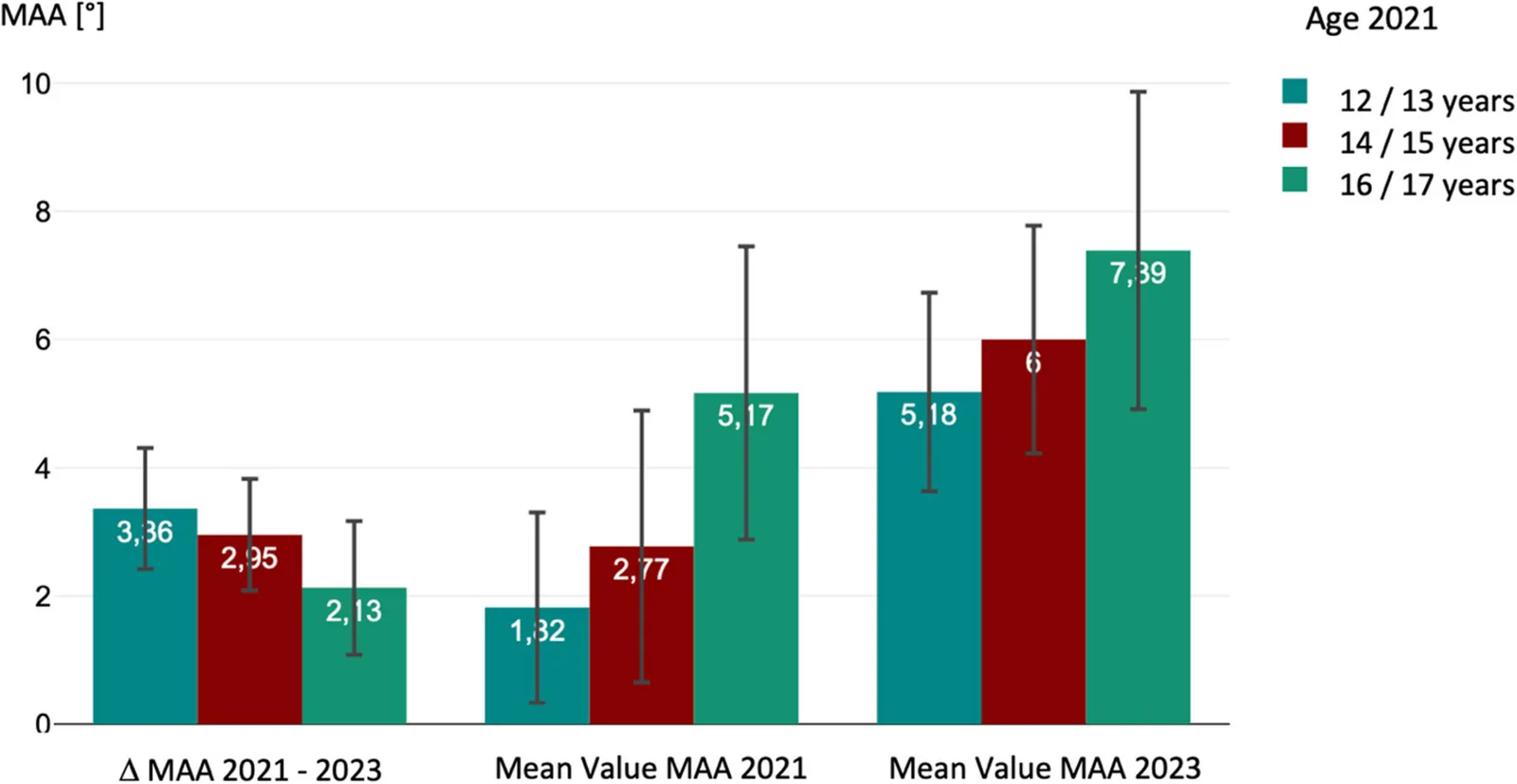
Graphic visualization of leg axis development between 2021 and 2023 by age group and depiction of differences between young adolensents (12/13 years), mid-adolescents (14/15 years) and old (16/17 years) athletes. (MAA: mechanical femorotibial axis angle, ∆: Delta / Difference between 2021 and 2023)

A significant association between age group at baseline and baseline MAA was observed (r(29) = 0.36, *p* = 0.049): the highest initial value in 2021 was 5.17 ± 3.50 degrees for subjects in the *older adolescents*. The lowest value was found in the *young adolescents* with an average MAA of 1.82 ± 2.51 degrees. The greatest increase in in mean MAA over the following years, with an average increase of 3.36 ± 1.60 degrees, was seen in *young adolescents* at baseline (1.82 ± 2.51 degrees in 2021 vs. 5.17 ± 3.50 degrees in 2023). The older adolescent group showed an average increase of only 2.22 ± 1.44 degrees (5.17 ± 3.50 degrees in 2021 vs. 7.39 ± 3.79 degrees in 2023) (Fig. 2)*.* An overview of the collected demographic data and measured values of the athletes by age group is shown in Table 1.

**Table 1:** Overview of the collected demographic data and measured values of the athletes by age group

	young adolescents(*n* = 11)	mid-adolescents(*n* = 11)	olderadolescents(*n* = 9)
Anthropometric variables			
Height 2021 [cm]	161.45 ± 9.97	174.55 ± 7.45	178.78 ± 8.21
Height 2023 [cm]	174.27 ± 7.04	179.27 ± 6.74	180.22 ± 8.01
∆ Height 2021–2023 [cm]	12.82 ± 5.47	4.73 ± 4.84	1.44 ± 0.88
Weight 2021 [kg]	48.45 ± 10.02	59.82 ± 8.05	69.22 ± 7.92
Weight 2023 [kg]	62.27 ± 8.89	68.36 ± 7.68	74.89 ± 5.78
∆ Weight 2021–2023 [kg]	13.82 ± 5.06	8.55 ± 3.80	5.67 ± 4.12
BMI 2021 [kg/m^2^]	18.38 ± 1.82	19.55 ± 1.42	21.69 ± 2.44
BMI 2023 [kg/m^2^]	20.48 ± 2.44	21.20 ± 1.24	23.07 ± 1.32
∆ BMI 2021–2023 [kg/m^2^]	2.10 ± 1.49	1.66 ± 0.79	1.39 ± 1.28
Lower-limb alignment variables			
Mean Value MAA 2021 [°]	1.82 ± 2.51	2.77 ± 3.59	5.17 ± 3.5
Mean Value MAA 2023 [°]	5.18 ± 2.62	6 ± 3.01	7.39 ± 3.79
∆ MAA 2021–2023 [°]	3.36 ± 1.6	2.95 ± 1.47	2.22 ± 1.44
MAA left leg 2021 [°]	1.36 ± 3.23	3.18 ± 4.12	5.11 ± 4.01
MAA right leg 2021 [°]	2.27 ± 2.65	2.36 ± 3.35	5.22 ± 3.27
Mean value MAA 2021 [°]	1.82 ± 2.51	2.77 ± 3.59	5.17 ± 3.50
MAA left leg 2023 [°]	4.73 ± 2.94	5.45 ± 3.33	6.78 ± 3.96
MAA right leg 2023 [°]	5.64 ± 2.87	6.55 ± 2.88	8.00 ± 3.71
Mean Value MAA 2023 [°]	5.18 ± 2.62	6.00 ± 0.31	7.39 ± 3.79
∆ MAA right leg 21–23 [°]	3.36 ± 2.73	4.09 ± 1.58	2.78 ± 2.17
∆ MAA left leg 21–23 [°]	3.36 ± 1.50	2.36 ± 2.16	1.67 ± 1.12

*MAA* Mechanical femorotibial axis angle, *BMI* Body-Mass-Index

∆: Delta / Difference between 2021 and 2023

A moderate negative association was observed between age at baseline and changes in MAA over the observation period (*r* = -0.33, *p* = 0.067), indicating that younger athletes tended to show larger increases in MAA than older athletes. In simple linear regression, age at baseline accounted for approximately 11.11% of the variance of the subsequent change in MAA after two years (F = 3.62, *p* = 0.067, R^2^ = 0.11, SDSF 1.47). Baseline MAA was significantly associated with subsequent changes in MAA (r(29) = -0.42, *p* = 0.017) and accounted for approximately 18% of the variance (F = 6.36, R^2^ = 0.18). Thus, greater baseline varus alignment was associated with a smaller increase in MAA during the observation period (Tables 2 and 3).

**Table 2:** Results of the linear regression analysis, which was used to analyze the influence of demographic variables on the change in the MAA over the observation period. With an R^2^ of 0.14, the regression model showed that the variable ‘height 2021’ explained 14% of the variance in the change in the MAA. For the prediction, the model has a standard error of 1.44. The effect deviates significantly from zero

Age 2021	*R*	*R* ^2^	adjusted *R*^2^	SD	df	F	*p*
	0.33	0.11	0.08	1.47	1	3.62	0.067
∆ Height	0.47	0.22	0.19	1.38	1	8.19	0.008
∆ Weight	0.55	0.3	0.27	1.31	1	12.29	0.002
∆ BMI	0.37	0.13	0.1	1.45	1	4.48	0.043
Height 2021	0.38	0.14	0.11	1.44	1	4.88	0.035
Weight 2021	0.5	0.25	0.23	1.35	1	9.72	0.004
BMI 2021	0.5	0.25	0.23	1.35	1	9.83	0.004
MAA 2021	0.42	0.18	0.15	1.41	1	6.36	0.017

*MAA* Mechanical femorotibial axis angle, *BMI* Body-Mass-Index

∆: Delta / Difference between 2021 and 2023

F = 4.88, *p* = 0.035

**Table 3:** Regression model and regression coefficients of the demographic data, which indicate the influence of the respective variables on the change in the MAA

model	unstandardized coefficient	standardized coefficient					95% confidence interval for B		
	B	Beta	SD		t	*p*	lower limit	upper limit	
C Age 2021	7.19 –0.3	-0.33	2.27 0.16		3.16 0.16	0.004 0.067	2.54 -0.62	11.83 0.02	
C ∆ Height	2.15 0.11	0.47	0.36 0.04		5.99 2.86	< 0.001 0.008	1.41 0.03	2.88 0.19	
C ∆ Weight	1.41 0.15	0.55	0.48 0.04		2.93 3.51	0.007 0.002	0.43 0.06	2.4 0.24	
C ∆ BMI	2.09 0.46	0.37	0.46 0.22		4.54 2.21	< 0.001 0.043	1.15 0.02	3.03 0.91	
C Height 2021	11.76 − 0.05	-0.38	4.02 0.02		2.92 -2.21	0.007 0.035	3.53 -0.1	19.98 0	
C Weight 2021	6.63 − 0.06	-0.5	1.22 0.02		5.41 -3.12	< 0.001 0.004	4.13 -0.11	9.13 -0.02	
C BMI 2021	9.57 − 0.34	-0.5	2.15 0.11		4.46 -3.14	< 0.001 0.004	5.18 -0.56	13.96 -0.12	
C MAA 2021	3.48 − 0.19	-0.42	0.35 0.08		10.05 -2.52	< 0.001 0.017	2.77 -0.35	4.19 -0.04	

*MAA *Mechanical femorotibial axis angle, *BMI* Body-Mass-Index

∆: Delta / Difference between 2021 and 2023

Both baseline height (171.13 ± 11.22 cm, *p* = 0.035) and height growth during the observation period (∆ Height; 6.65 ± 6.44 cm, *p* = 0.008) were significantly associated with changes in MAA. Baseline height was negatively associated with changes in MAA (r(29) = − 0.38, *p* = 0.035), whereas ∆ Height was positively associated with changes in MAA (r(29) = 0.47, *p* = 0.008). Thus, greater increases in height during the observation period were associated with larger increases in varus alignment.

As expected, younger athletes were shorter at baseline and showed substantially greater increases in height over the observation period than older athletes. These greater increases in height coincided with larger increases in MAA. Simple linear regression showed that baseline height accounted for approximately 14% of the variance in MAA change (F = 4.88, p = 0.035, R² = 0.14), whereas ΔHeight accounted for approximately 22% (F = 8.19, p = 0.008, R² = 0.22). A statistically significant positive correlation was also found between the increase in MAA and body weight (r(29) = 0.55, p = 0.002) and BMI (r(29) = 0.37, p = 0.043) (Table 4).

**Table 4:** Results of the correlation analysis with the demographic variables measured for the first time in 2021 (age, height, weight, BMI) and their change over the 24-month observation period with the change in the MAA

	Age 2021	∆ Height	∆ Weight	∆ BMI	Height 2021	Weight 2021	BMI 2021	MAA 2021
*r*	-0.33	0.47	0.55	0.37	-0.38	-0.5	-0.5	-0.42
p	0.067	0.008	0.002	0.043	0.035	0.004	0.004	0.017

*MAA* Mechanical femorotibial axis angle, *BMI* Body-Mass-Index

∆: Delta / Difference between 2021 and 2023

Multiple linear regression analyses were performed to examine the combined associations of age and anthropometric variables with changes in MAA. The exploratory multivariable model including age at baseline and baseline height explained 15.95% of the variance in MAA change but did not reach statistical significance (F = 2.66, *p* = 0.086, R² = 0.16). Baseline height showed the stronger standardized regression coefficient (β = -0.28 vs. -0.16).

The primary multivariable model including age at baseline and ΔHeight showed a significant association with changes in MAA (F = 3.95, *p* = 0.03, R² = 0.22), with ΔHeight emerging as the stronger predictor. Additional exploratory multivariable analyses are presented in Supplementary Tables S1 and S2.

No significant association was observed between dominant kicking leg and overall changes in MAA (*p* = 0.282) or side-specific alignment changes of either the right (*p* = 0.385) or left leg (*p* = 0.632).

## Discussion

The main finding of this longitudinal study was that, within this selected cohort of adolescent academy football players, greater increases in height and associated weight gain were most strongly associated with greater progression of varus alignment over the two-year observation period. To the best of our knowledge, this is one of the first longitudinal studies to follow changes in varus alignment in the same cohort of adolescent football players over a two-year period. Within this cohort, younger athletes tended to show greater increases in MAA than older athletes. These findings suggest that varus progression may be more pronounced during early adolescence than during later stages of adolescence.

During physiological leg axis development, natural valgus alignment reaches its maximum at the age of 3–5 years, corrects itself spontaneously by the age of 10 years and persists into adulthood [23, 24]. The results of this study, however, demonstrate that the leg axis in a cohort of highly active football players can further change during adolescence. Some studies suggest that the practice of bone-strengthening sports during the growth phase causes an increasing varus alignment of the leg axes [1–3, 6] Evidence suggests that repetitive stress from intensive training—particularly activities involving weight-bearing, twisting, and rapid starting and stopping—places increased compression on the physis of the young, immature skeleton. This growing structure may be particularly vulnerable to maladaptation from uneven compressive forces during periods of rapid skeletal growth [25, 26] According to the law of Hueter-Volkmann et al., this increased compression at the physis may lead to delayed bone growth or growth arrest with consequent deformity of the leg axis on the affected side [2, 3, 27, 28] Repeated microtraumas, increased anterior cruciate ligament tension and strong pes anserinus contractions may exacerbate the effect [1, 3, 4, 6, 29]. De Cock et al. observed varus deformity mainly in athletically active participants during adolescence [2,30]. De Thijs et al. and Steffen et al. further suggested that football players between approximately 13 and 15 years of age may represent a particularly vulnerable subgroup [3]. However, all of these studies were cross-sectional in design and therefore do not permit conclusions regarding causal relationships. Based on the results of this longitudinal study, greater increases in height and weight were associated with larger increases in varus alignment and may indicate a potentially vulnerable developmental period in active football players. According to Stratton et al., a potentially vulnerable developmental period for changes in knee alignment may occur between the ages of 12 and 17 years [2, 3, 26] The present findings describe associations observed over a two-year period and do not permit identification of the exact timing of peak height velocity or other maturation milestones. However, these findings should be interpreted as observational associations rather than causal effects. The overall increase in varus alignement was greater in the younger athletes (3.36 ± 1.60°) than in the older athletes (2.22 ± 1.44°). Therefore, the present findings support the hypothesis that younger adolescent football players may experience greater progression of varus alignment during periods of more rapid growth, whereas changes appear to become smaller during later adolescence [31].

An influence of the shooting or the stance leg could not be detected in this study, supporting the results of Chantraine et al [1, 32]. Therefore, the mean MAA of both legs was retained as the primary outcome measure in the present study to characterise overall lower-limb alignment rather than side-specific adaptations. Future studies should investigate whether different playing positions, with their specific movement demands and loading characteristics (e.g. high-frequency dodging, cutting and turning movements in the forward position), are associated with different patterns of varus progression.

Variation of the leg axis implies a shift of the main mechanical load axis to the medial knee compartment and increases the risk of cartilage damage and the manifestation of medial knee osteoarthritis [15, 33–36]. Brouwer et al. estimate that the risk of developing osteoarthritis of the knee joint is doubled in the presence of genu varum [37] The present findings may help to inform the timing of future preventive approaches, particularly during periods of pronounced growth. However, the effectiveness of specific preventive interventions, such as leg-axis stabilisation exercises, biomechanical training approaches, or orthotic interventions, remains unknown and should be evaluated in prospective interventional studies.

### Limitations

The limitations of the study include the relatively small sample size, which may have limited statistical power, particularly for multivariable analyses, and the heterogeneous ethnic background of the participants, which was not recorded. In addition, all athletes originated from a single professional youth academy with comparable training philosophy, load management, and medical care. Detailed information regarding weekly training volume, match exposure, playing position, previous injury history, and formal athlete classification frameworks was not systematically recorded and may represent additional factors influencing lower-limb development. Future studies should incorporate these variables to improve the characterisation of young football cohorts. While this ensured a high degree of internal consistency, the findings may not be directly transferable to other football populations. Moreover, more detailed anthropometric measures, including lower-limb length, limb-length asymmetry, and segmental measurements of the thigh and shank, were not available. Such variables may contribute to a more comprehensive understanding of the relationship between growth patterns and changes in lower-limb alignment and should be considered in future studies.

Additionally, excluding athletes without varus progression may have introduced selection bias and potentially overestimated the observed associations between anthropometric changes and increasing varus alignment. Therefore, the findings should primarily be interpreted within the selected cohort of male academy football players demonstrating varus progression and may not be generalisable to female athletes, recreational players, or athletes from other sports disciplines.

Likewise, biological maturation was not directly assessed using established indicators such as peak height velocity, Tanner stage, or skeletal age. Therefore, chronological age and changes in body height were used as surrogate measures, which may not fully reflect individual differences in maturation status. Future longitudinal studies should incorporate direct assessments of biological maturity to better distinguish the influence of maturation status, chronological age, and training exposure on leg-axis development. Finally, due to the observational study design, residual confounding cannot be excluded, and the reported associations should not be interpreted as evidence of causality. In addition, the proportion of variance explained by the regression models was modest, and several analyses should be considered exploratory. Furthermore, study-specific estimates of intra-rater reliability, inter-rater reliability, and measurement error (e.g., SEM or MDC) were not determined. In addition, formal blinding to previous assessments was not implemented, although the linked dataset was only created after completion of data collection.

## Conclusion

The main finding of this longitudinal study was that, within this selected cohort of academy football players, greater increases in height and associated weight gain were most strongly associated with progression of varus alignment over the two-year observation period. The increase in MAA over the two-year observation period was most pronounced in younger athletes (12–13 years at baseline). These findings may help to identify developmental periods during which preventive strategies could be evaluated in future studies. The potential impact of specific interventions on leg-axis development, however, remains to be established.

## Supplementary Information

Supplementary Material 1.

## Data Availability

The datasets used and/or analysed during the current study are available from the corresponding author on reasonable request.

## Abbreviations

ACL: Anterior cruciate ligament
BMI: Body mass index
e.g.: Exempli gratia / for example
MAA: Mechanical femorotibial axis angle
MRI: Magnetic Resonance Imaging
PHV: Peak height velocity
