# Supplementary material for: Two-year follow-up of varus progression in young professional football players
Source: BMC Sports Sci Med Rehabil. 2026 Sep 11;18:420. doi: 10.1186/s13102-026-02035-7 (PMC13576229; doi:10.1186/s13102-026-02035-7)
Supplement: Supplementary file 1 [file 13102_2026_2035_MOESM1_ESM.pdf]

Supplementary Material

The supplementary analyses presented below provide additional exploratory multivariable regression models investigating potential associations between demographic characteristics, anthropometric measures, baseline lower-limb alignment, and changes in the mechanical femorotibial axis angle (MAA) over the 24-month observation period. In accordance with the a priori study hypothesis, the primary analyses reported in the main manuscript focused on chronological age at baseline and changes in body height (Δ Height). The models included in this supplement are therefore intended as secondary exploratory analyses and should be interpreted cautiously considering the modest sample size and the observational study design. They are provided to ensure transparency and to allow a more comprehensive assessment of potential interacting factors influencing varus progression in adolescent male academy football players.

TABLE S1

	R	R^2	adjusted R	SD	df	F	p
**Age + Height 2021**	0.4	0.16	0.10	1.45	2	2.66	0.085
**Height + MAA 2021**	0.51	0.26	0.21	1.36	2	4.94	0.014
**Age 2021 + ∆ Height**	0.47	0.22	0.16	1.4	2	3.95	0.03
**∆ Height + MAA 2021**	0.51	0.26	0.21	1.36	2	4.92	0.014
**2021: Age, Height, Weight**	0.53	0.28	0.20	1.37	2	3.51	0.027
**2021: Age, Height, MAA**	0.51	0.26	0.18	1.39	2	3.20	0.037
**2021: Age, MAA, ∆ Height**	0.51	0.26	0.18	1.39	2	3.17	0.038

Table S1: Part 1; Results of the multiple linear regression analysis carried out to investigate the influence of various combined variables (e.g. age 2021 and height 2021) on the increasing variation of the MAA over the observation period. MAA: mechanical femorotibial axis angle, BMI: Body-Mass-Index, ∆: Delta / Difference between 2021 and 2023)

TABLE S2

	unstandardized coefficient	standardized coefficient				95% confidence interval for B	
**model**	**B**	**Beta**	**SD**	**t**	**p**	**lower limit**	**upper limit**
**C**	11.5		4.07	2.82	0.009	3.16	19.84
**Age 2021**	-0.14	-0.16	0.20	-0.72	0.479	-0.55	0.27
**Height 2021**	-0.04	-0.28	0.03	-1.27	0.215	-0.1	0.02
**C**	10.24		3.87	2.65	0.013	2.32	18.17
**Height 2021**	-0.04	-0.29	0.02	-1.75	0.091	-0.09	0.01
**MAA 2021**	-0.16	-0.35	0.06	-2.10	0.044	-0.31	0
**C**	2.1		3.36	0.63	0.536	-4.78	8.98
**Age 2021**	0	0	0.21	0.01	0.99	-0.44	0.44
**Δ Height**	0.11	0.47	0.06	1.98	0.058	0	0.23
**C**	2.69		0.57	4.75	<0.001	1.53	3.85
**Δ Height**	0.08	0.34	0.05	11.74	0.092	-0.01	0.18
**MAA 2021**	-0.11	-0.24	0.09	-1.23	0.228	-0.29	0.07
**C**	-0.17		6.69	-0.03	0.96	-13.9	13.56
**Age 2021**	0.14	0.15	0.23	0.6	0.556	-0.33	0.61
**Height 2021**	0.05	0.34	0.05	0.95	0.351	-0.05	0.15
**Weight 2021**	-0.12	-0.92	0.06	-2.13	0.042	-0.23	0

**C**	10.21		3.94	2.59	0.015	2.12	18.3
**Age 2021**	-0.04	-0.04	0.20	-0.20	0.843	-0.45	0.37
**Weight 2021**	-0.04	-0.27	0.03	-1.27	0.214	-0.1	0.02
**MAA 2021**	-0.15	-0.34	0.06	-1.94	0.063	-0.32	0.01
**C**	2.87		3.39	0.85	0.405	-4.09	9.83
**Age 2021**	-0.01	-0.01	0.21	-0.06	0.956	-0.45	0.43
**∆ Height**	0.08	0.33	0.06	1.25	0.223	-0.05	0.21
**MAA 2021**	-0.11	-0.24	0.09	-1.21	0.236	-0.29	0.07

Table S2: Part 2; Results of the multiple linear regression analysis carried out to investigate the influence of various combined variables (e.g. age 2021 and height 2021) on the increasing variation of the MAA over the observation period. MAA: mechanical femorotibial axis angle, BMI: Body-Mass-Index, ∆: Delta / Difference between 2021 and 2023)
